# SVM-enhanced attention mechanisms for motor imagery EEG classification in brain-computer interfaces

**DOI:** 10.3389/fnins.2025.1622847

**Published:** 2025-07-11

**Authors:** Zhenis Otarbay, Abzal Kyzyrkanov

**Affiliations:** ^1^Department of Science and Innovation, Astana IT University, Astana, Kazakhstan; ^2^Department of Robotics, School of Engineering and Digital Sciences, Nazarbayev University, Astana, Kazakhstan

**Keywords:** brain-computer interface, motor imagery, EEG classification, deep learning, convolutional neural network, long short-term memory, self-attention mechanism, support vector machine

## Abstract

Brain-Computer Interfaces (BCIs) leverage brain signals to facilitate communication and control, particularly benefiting individuals with motor impairments. Motor imagery (MI)-based BCIs, utilizing non-invasive electroencephalography (EEG), face challenges due to high signal variability, noise, and class overlap. Deep learning architectures, such as CNNs and LSTMs, have improved EEG classification but still struggle to fully capture discriminative features for overlapping motor imagery classes. This study introduces a hybrid deep neural architecture that integrates Convolutional Neural Networks, Long Short-Term Memory networks, and a novel SVM-enhanced attention mechanism. The proposed method embeds the margin maximization objective of Support Vector Machines directly into the self-attention computation to improve interclass separability during feature learning. We evaluate our model on four benchmark datasets: Physionet, Weibo, BCI Competition IV 2a, and 2b, using a Leave-One-Subject-Out (LOSO) protocol to ensure robustness and generalizability. Results demonstrate consistent improvements in classification accuracy, F1-score, and sensitivity compared to conventional attention mechanisms and baseline CNN-LSTM models. Additionally, the model significantly reduces computational cost, supporting real-time BCI applications. Our findings highlight the potential of SVM-enhanced attention to improve EEG decoding performance by enforcing feature relevance and geometric class separability simultaneously.

## 1 Introduction

BCIs are devices that can circumvent traditional communication channels (such as muscles and speech), converting various images of activity of the brain to instructions, allowing direct communication between the human cortex and external devices (Millan et al., 2010). People with ALS and Parkinson's disease may require the BCI-assisted system for communication. BCI can be used to send the signal directly without needing any muscle activity. This paper applies batch normalization (BN) within a CNN framework to solve the over-fitting problem. We use ReLU activation in convolutional layers to accelerate the training duration. Batch normalization improves classification performance with fewer training epochs. Signal recordings of brain activity used by BCIs can be either invasive or non-invasive. Invasive BCIs require surgical intervention to implant electrodes directly on or inside the cortex, whereas non-invasive BCIs do not require surgical manipulations. Non-invasive BCIs can use various brain signals as inputs, such as electroencephalograms (EEG), magnetoencephalograms (MEG), blood-oxygen-level-dependent (BOLD) signals, and (de) oxyhemoglobin concentrations (Nijholt et al., 2008). EEG is preferred due to its high temporal resolution, safety, and low cost. It does not need any invasion (Sundararajan et al., 2015), although it is still necessary to develop alternative interfaces that allow disabled people to use EEG for communication with autonomous systems.

An EEG signal known as motor imaging (MI) relates to brain signals generated by visualizing limb movement but not natural limb movement (Al-Saegh et al., 2021). Analyzing the MI signal makes it possible to judge the imaginary movement intention and operate the external device. Eventually, motor imagery control has significant potential for applications such as in various fields, such as recreational activity rehabilitation function, motor assistance function, etc.

Therefore, the MI signal has become one of the most commonly used signals in the BCI. However, EEG classification is challenging because of non-stationary EEG signals and the influence of many background waveforms and artifacts. To address these challenges, this study focuses on integrating SVM within the attention mechanism to improve EEG classification. While attention mechanisms help highlight relevant EEG features, they do not inherently optimize class separability.

Despite recent advancements in EEG classification, existing methods still struggle with subtle and overlapping patterns, which are common in motor imagery tasks. CNNs, widely used for feature extraction, are effective at capturing spatial structures but often fail to model long-range dependencies, making them insufficient for complex temporal variations. Long Short-Term Memory (LSTM) networks, on the other hand, excel at modeling temporal dependencies but can struggle with high-dimensional EEG data, leading to suboptimal feature extraction and difficulty in distinguishing overlapping motor imagery patterns. Attention mechanisms have been introduced to address these issues, but standard attention lacks the ability to explicitly enforce class separation. This limitation reduces their effectiveness in distinguishing closely related motor imagery classes, especially when EEG signals exhibit significant overlap. This highlights a gap in current approaches—where a combination of attention mechanisms and margin-based learning techniques, such as SVM, could provide a more effective solution. To address this gap, we propose integrating SVM's margin-maximization principle into the attention mechanism to simultaneously support feature relevance and class boundary separation. This integration ensures that overlapping EEG features are better distinguished, leading to improved classification performance and robustness against noise.

Classifying motor imagery EEG data presents significant challenges due to the high dimensionality, inherent noise, and overlapping signal patterns in EEG recordings. These characteristics make it difficult for standard classification methods to identify clear, distinct patterns, leading to reduced accuracy in motor imagery tasks. Attention mechanisms have emerged as a promising approach to tackle this complexity by allowing the model to selectively focus on relevant features, thus enhancing the representation of task-specific patterns in noisy data. However, traditional attention models focus on feature weighting but lack explicit optimization for class boundaries, which is essential for distinguishing motor imagery tasks in noisy EEG data. This combination enhances the model's robustness and accuracy in motor imagery classification, especially in dealing with overlapping features typical of EEG signals.

Nowadays, almost all Motor Imagery BCI (MI-BCI) systems summarize the most relevant information about the measurements in two kinds of covariance matrices: the covariance matrices of the filtered observations employed for dimensionality reduction and the covariance matrices of the features required for classification purposes. In the first stage of the dimension reduction technique, we select those sub-spaces of the observations that retain most of the discriminative powers. We can, for example, employ (CSP) to MI EEG data (Pfurtscheller et al., 1991).

The use of DL for the categorization of MI EEG data primarily concerns the following issues:

- what are the most effective model selection procedures for deep learning categorization of MI EEG data?
- which input data format has the most significant influence on the deep learning system?
- what frequency range should be considered throughout the analysis?

The comprehensive review by Al-Saegh et al. (2021) summarizes major aspects of motor imagery EEG classification, including benchmark datasets, deep neural network (DNN) architectures, key frequency bands, regularization strategies, and preprocessing techniques commonly used in the field.

The usage of EEG signals in motor imagery tasks suffers from poor spatial resolution due to the volumetric calculation effects. It may result in a not perfectly accurate design and application of BCI. This paper introduces the framework with sparse spectrotemporal decomposition. It is a CNN architecture with improved classification accuracy in terms of accuracy, and kappa value, squeeze, and excitation (SE) blocks. The channels are re-calibrated more precisely.

This study seeks to answer the following research question: can integrating SVM constraints within the attention mechanism enhance EEG classification by improving class separability, refining feature representation, and increasing robustness against noise in motor imagery tasks? Successfully addressing this question would contribute to the development of more accurate and reliable EEG-based classification models, which are essential for real-world BCI applications.

## 2 Related work

Attention mechanisms have become crucial in EEG classification, enhancing feature selection by focusing on task-relevant EEG patterns in noisy data. Recent studies have applied attention-based models to EEG tasks such as, including music-induced emotion recognition, motor imagery, and multimodal EEG analysis (Wang et al., 2024; Pichandi et al., 2024; Gao et al., 2024). For example, Wang et al. employed a bidirectional LSTM with attention to enhance EEG-based music-induced emotional state recognition, where the model selectively emphasizes key EEG features relevant to emotion (Wang et al., 2024). Similarly, Pichandi et al. introduced a hybrid attention-based deep learning model for parallel feature extraction, improving the classification of EEG signals related to emotional states (Pichandi et al., 2024). These studies underscore the versatility of attention in EEG applications, particularly in handling high-dimensional data. Building on this, recent works have demonstrated the effectiveness of hybrid attention mechanisms in motor imagery, depression diagnosis, and multimodal EEG analysis.

Recent advancements in attention-based models have further improved EEG classification performance. Liu and Huang (2024) introduced DualDomain-AttenNet, a hybrid deep learning model that synergizes time-frequency analysis with attention mechanisms to enhance motor imagery EEG classification (Liu and Huang, 2024). Similarly, Gao et al. (2024) developed a multiscale feature fusion network integrating attention mechanisms, which significantly improved the decoding of motor imagery EEG data by focusing on relevant spatial and temporal features (Gao et al., 2024). Wang et al. (2022) explored hybrid neural networks with attention mechanisms for depression diagnosis, demonstrating that attention-enhanced models can extract meaningful EEG features even from complex clinical datasets (Wang et al., 2022).

Moreover, Hybrid models combining SVM with attention mechanisms have shown promise for handling high-dimensional EEG data. Recent research has explored hybrid models that integrate SVM with deep learning techniques to refine EEG feature separability, particularly in unsupervised and sparse representation learning. Tanwar et al.'s wearable-based stress recognition model incorporates SVM alongside attention layers, enabling the model to focus on stress-related features within complex EEG signals, thus enhancing classification accuracy (Tanwar et al., 2024). In another study, Liu et al. combined an attention mechanism with an SVM-based convolutional capsule network to improve emotion recognition accuracy, particularly in high-dimensional EEG data classification tasks (Liu et al., 2023).

In addition to stress and emotion recognition, hybrid SVM models have been explored for various EEG classification tasks. Liang et al. (2021) introduced EEGFuseNet, a hybrid deep learning approach that integrates unsupervised feature characterization with SVM-based classifiers to enhance high-dimensional EEG classification (Liang et al., 2021). Similarly, Prabhakar and Lee (2022) developed a sparse representation-based hybrid model, combining deep learning with SVM to improve EEG signal robustness against noise (Prabhakar and Lee, 2022). These studies demonstrate the potential of SVM-based hybrid models in EEG classification, particularly in improving class separability and robustness. However, they primarily use SVM for feature selection or post-processing, rather than fully embedding its optimization principles within deep learning architectures. This indicates a fundamental gap in the development of hybrid deep learning models—existing methods fail to integrate SVM's margin-maximization properties directly into attention mechanisms, which are crucial for refining class separability in EEG classification.

Although SVM has shown strong performance in high-dimensional EEG tasks, its integration into deep networks remains limited. Most existing approaches either employ SVM as a standalone classifier or use it for feature selection without fully embedding its optimization principles within deep networks. Liang et al. (2021) and Prabhakar and Lee (2022) demonstrated the feasibility of SVM-hybrid models, yet these implementations primarily rely on conventional feature extraction rather than incorporating SVM constraints into deep learning layers (Liang et al., 2021; Prabhakar and Lee, 2022). The lack of a structured approach to integrate SVM's margin-maximization capability directly into attention mechanisms presents a significant research gap.

In motor imagery tasks, attention mechanisms have proven valuable. Gao et al. developed a multiscale feature fusion network incorporating attention to decode motor imagery signals in EEG data, achieving high classification accuracy by emphasizing relevant motor imagery features (Gao et al., 2024). Similarly, Ma et al. used attention mechanisms within a CNN-BI-LSTM model to enhance seizure prediction, allowing the model to focus on seizure-related features in multi-channel EEG data (Ma et al., 2023).

Multimodal approaches also benefit from attention mechanisms. For instance, Cao et al. designed a classroom fatigue recognition model based on self-attention, which fuses EEG with other physiological signals. This approach effectively handles high-dimensional data and ensures robust performance by selectively emphasizing significant EEG features related to fatigue detection (Cao et al., 2024). These recent advancements highlight the adaptability and effectiveness of attention mechanisms in EEG classification, especially when combined with SVM in hybrid models to tackle high-dimensional challenges. Tao et al. introduced the Gated Transformer architecture to apply EEG signals decoded from the human brain signals (Tao et al., 2021).

## 3 Methods

To address the limitations identified in previous work, this study proposes a novel hybrid architecture that enhances EEG classification by explicitly optimizing for class separability. In motor imagery tasks, EEG signals are inherently noisy, non-stationary, and often exhibit significant overlap between classes. While attention mechanisms have proven effective at focusing on task-relevant features, they do not inherently enforce inter-class margin constraints during learning. As a result, even with attention, classification performance can suffer in the presence of overlapping features.

To overcome this challenge, we introduce an SVM-enhanced attention mechanism that integrates the margin-maximization principle of Support Vector Machines (SVM) directly into the attention computation. By embedding SVM optimization constraints within the attention layer, our approach not only captures feature relevance but also promotes the geometric separation of classes in the feature space. This dual objective leads to more robust decision boundaries and improved performance in noisy EEG environments.

Unlike previous hybrid models that use SVM in post-processing or as a standalone classifier, our method incorporates the margin-based formulation into the deep learning pipeline. Specifically, the SVM constraints are embedded within the self-attention mechanism, ensuring that feature selection and class separability are jointly optimized during training. The following subsections describe the data preprocessing steps, neural network architecture, and the implementation of the SVM-enhanced attention module.

### 3.1 Datasets

This study utilizes four publicly available EEG datasets widely used in motor imagery classification research. Each dataset includes labeled EEG signals recorded during imagined limb movements. Our experiments were conducted using binary classification tasks (e.g., left-hand vs. right-hand imagery) to ensure consistency across datasets. All trials were segmented into 4-s epochs following the motor imagery cue, and only sessions involving right and left-hand imagery were retained for model training and evaluation.

**Weibo 2014** dataset consists of EEG recordings from ten healthy, right-handed individuals (three female, seven male) aged between 23 and 25 years (Yi et al., 2014). A Neuroscan SynAmps2 amplifier was used to record EEG signals at 1,000 Hz, which were subsequently downsampled to 200 Hz. Participants were visually cued to imagine performing either left- or right-hand movements. Each subject completed nine sessions with 60 trials per session, totaling 540 trials per participant. The dataset was designed to examine differences between simple and compound motor imagery.

**PhysioNet** dataset, sourced from the PhysioBank repository, contains EEG recordings from 109 participants who performed various motor imagery tasks (Goldberger et al., 2000). The signals were acquired at a sampling rate of 160 Hz. For consistency with other datasets, we selected only the trials involving left- and right-hand imagery. Each subject performed 46 trials.

**BCI Competition IV dataset 2a** (referred to as BCI-IV 2a) dataset includes EEG data from nine subjects instructed to imagine four movements: left hand, right hand, both feet, and tongue (Tangermann et al., 2012). EEG was recorded using 22 Ag/AgCl electrodes at a sampling rate of 250 Hz, filtered between 0.5 and 100 Hz. For this study, only left- and right-hand imagery trials were used.

**BCI Competition IV dataset 2b** (referred to as BCI-IV 2b) also contains recordings from nine participants performing left- and right-hand motor imagery tasks (Leeb et al., 2007). EEG signals were recorded at 250 Hz and filtered in the 0.5-100 Hz range. Each subject completed five sessions. During the first two sessions, visual feedback was provided via an animated smiling face; the final three sessions were conducted without feedback.

### 3.2 Prepossessing of raw data

Raw EEG signals usually contain undesirable background noise, such as eye blinks requiring elimination before beginning the fundamental analysis. Furthermore, augmenting the raw EEG to meet the needs is occasionally helpful. It is possible to use one or more preprocessing procedures (Somers et al., 2018) might be applied.

**Figure 1:**
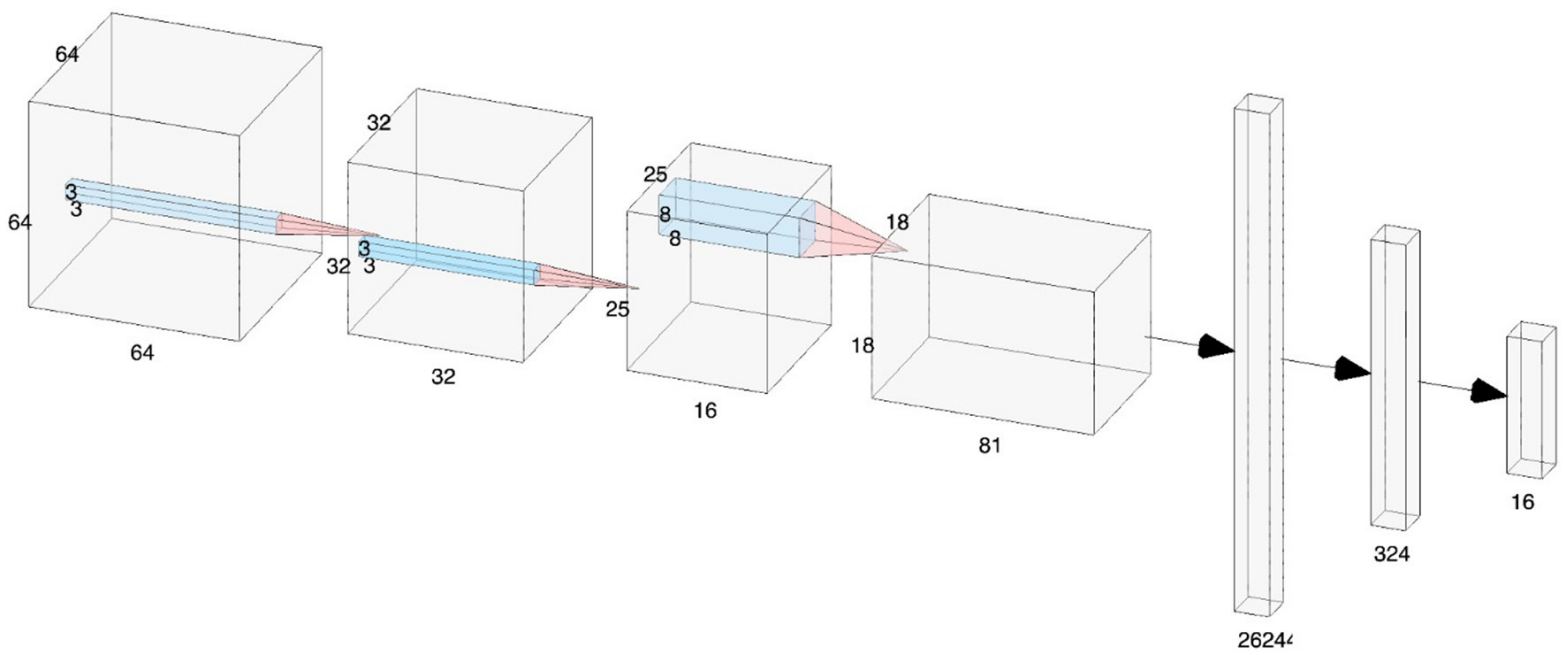
In-depth architecture of convolutional neural networks for EEG signal decoding in BCIs applications.

The deep learning research has shown enhanced performance in learning from raw EEG data, mitigating the need for preprocessing or handcrafted features (Zhang et al., 2020; Schirrmeister et al., 2017; Craik et al., 2019). Here, we apply minimal preprocessing to all four datasets, using a 4 Hz high-pass filter to suppress low-frequency noise while retaining informative signal components, and perform basic artifact rejection through statistical thresholding (Schirrmeister et al., 2017; Lawhern et al., 2018). The overall architecture of the CNN used for feature extraction is illustrated in Figure 1.

In this regard, the EEG waveforms were high-pass filtered above 4 Hz using a fourth-order Butterworth IIR filter. The a high-pass filter with a 4 Hz cut-off frequency was used to suppress electro-oculographic artifacts that arose due to eye movement dominant between 0.1 and 4 Hz band in EEG.

Other than that, and as it was suggested by Schirrmeister et al. (2017), we did not apply low-pass filtering to leave the raw EEG data intact.

Further, the continuous EEG was segmented into a lefthand and right-hand imagination trial with a 4-s length following the motor imagery onset. Subsequently, EEG data trials were artifact corrected by applying a statistical threshold to exclude: (i) bad EEG trials correlated with egregious movement noise; and (ii) channels that are noisy because of possible poor connection to the scalp of a participant. Bad trials were identified by calculating the mean absolute value per trial and eliminating trials with values higher than three standard deviations over the mean trial.

The preprocessing pipeline described above was applied consistently across all datasets to ensure comparability. This includes the use of a 4 Hz high-pass filter, artifact rejection via statistical thresholding, and trial segmentation into 4-s windows following motor imagery onset. No dataset-specific adjustments or alternative filtering procedures were introduced, allowing the evaluation to focus purely on model performance rather than differences in data preparation.

### 3.3 Deep neural networks architecture

We have implemented an approach that differs from that of Abibullaev et al. (2020), where we do not create separate depth dimensions for the input data but instead use the EEG channel as the CNN depth dimension. As with colors in RGB images, some channels are correlated, and some are linearly uncorrelated. We increase the depth to extract more features of EEG channels. Using EEG channels as depth allows us not to create a new dimension and to decrease the size of the output layer used as an input for a many-to-one LSTM layer and following a fully connected layer. By doing so, we increase computation speed by maintaining comparable results. Our approach can be used for real-time applications. The example for 3 EEG channels as convolution layer's depth (channels) and depth dimension 3:9:18 is increased with kernel size 1 × 8.

**Figure 2:**
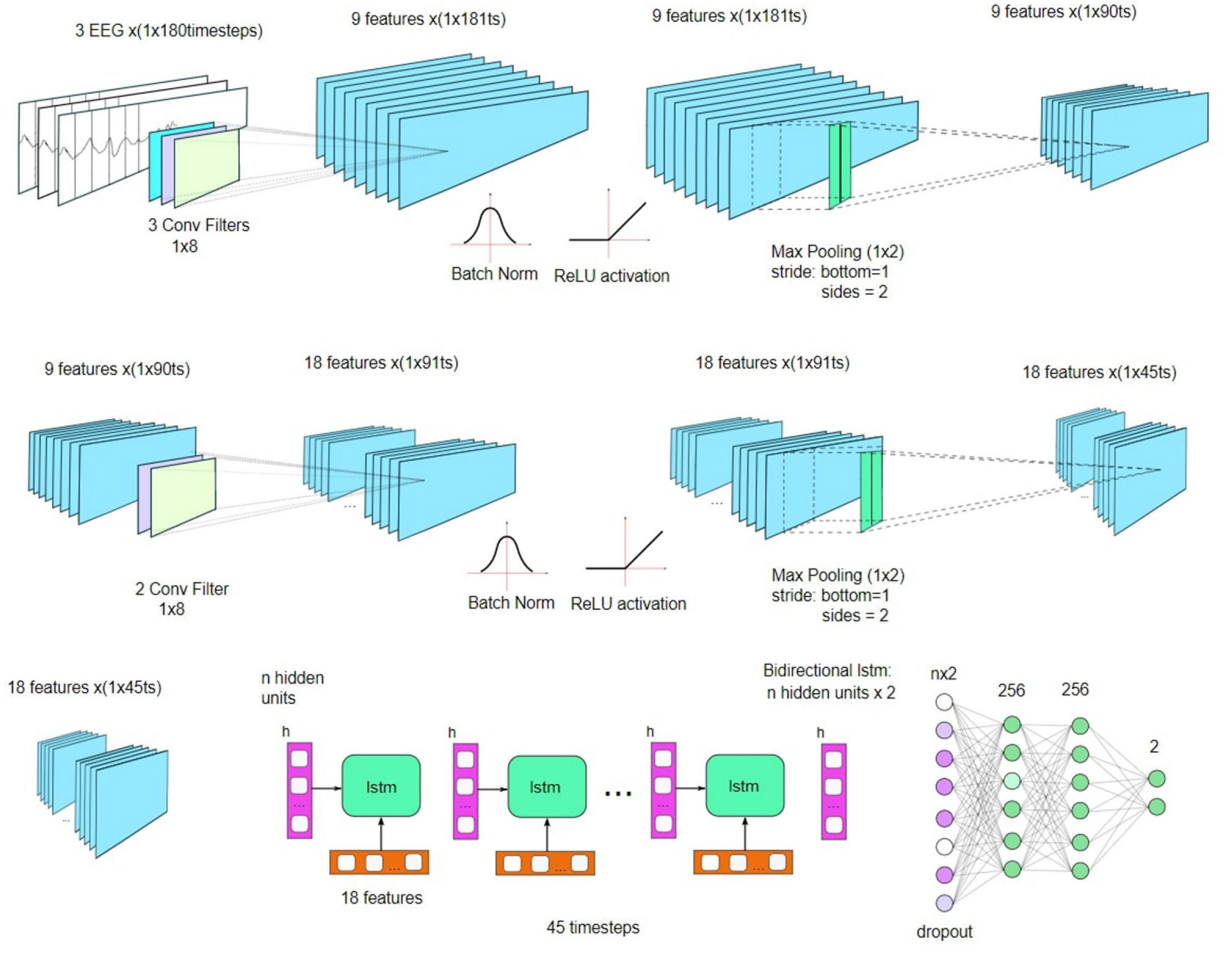
Detailed architecture of the CNN and bidirectional LSTM model used for motor imagery EEG classification across all datasets.

We tested this architecture with kernels (1 × 8), (1 × 24), and (1 × 40). Instead of increasing depth in geometric progression (*chans*, *chans*^2^, *chans*^3^ ...), we used algebraic progression (*chans*, *chans*×2, *chans*×3 ...), as shown in Figure 2, which further decreased time spent on CNN computation. The embedded bidirectional LSTM layer uses samples over the timespan (LSTM units) and EEG or CNN channels as input features. Hidden units consisted of 128 nodes for Physionet and Weibo and 256 for BCI IV 2a and BCI IV 2b. We started from a higher CNN depth compared to the original approach (Abibullaev et al., 2020) to reduce the input length to the LSTM layer. The overall architecture first extracts spatial features using CNN, then models temporal dependencies through the bidirectional LSTM, and finally applies an SVM-enhanced attention mechanism to reweight the LSTM outputs, promoting features that maximize class separability before passing them to the final classification layer.

### 3.4 Transformer networks for BCI IV 2b and Physionet

Transformer networks are based on an attention mechanism and allow GPUs to run in parallel. We can activate or deactivate the CNN by changing the ConvDOWN boolean. Moreover, it is possible to crop the data for suitable deep learning classification. All data are concatenated, and 45-length crops are used if there is more than one file. The input images are resized to 72, and the patch size is extracted from the input data.

**Figure 3:**
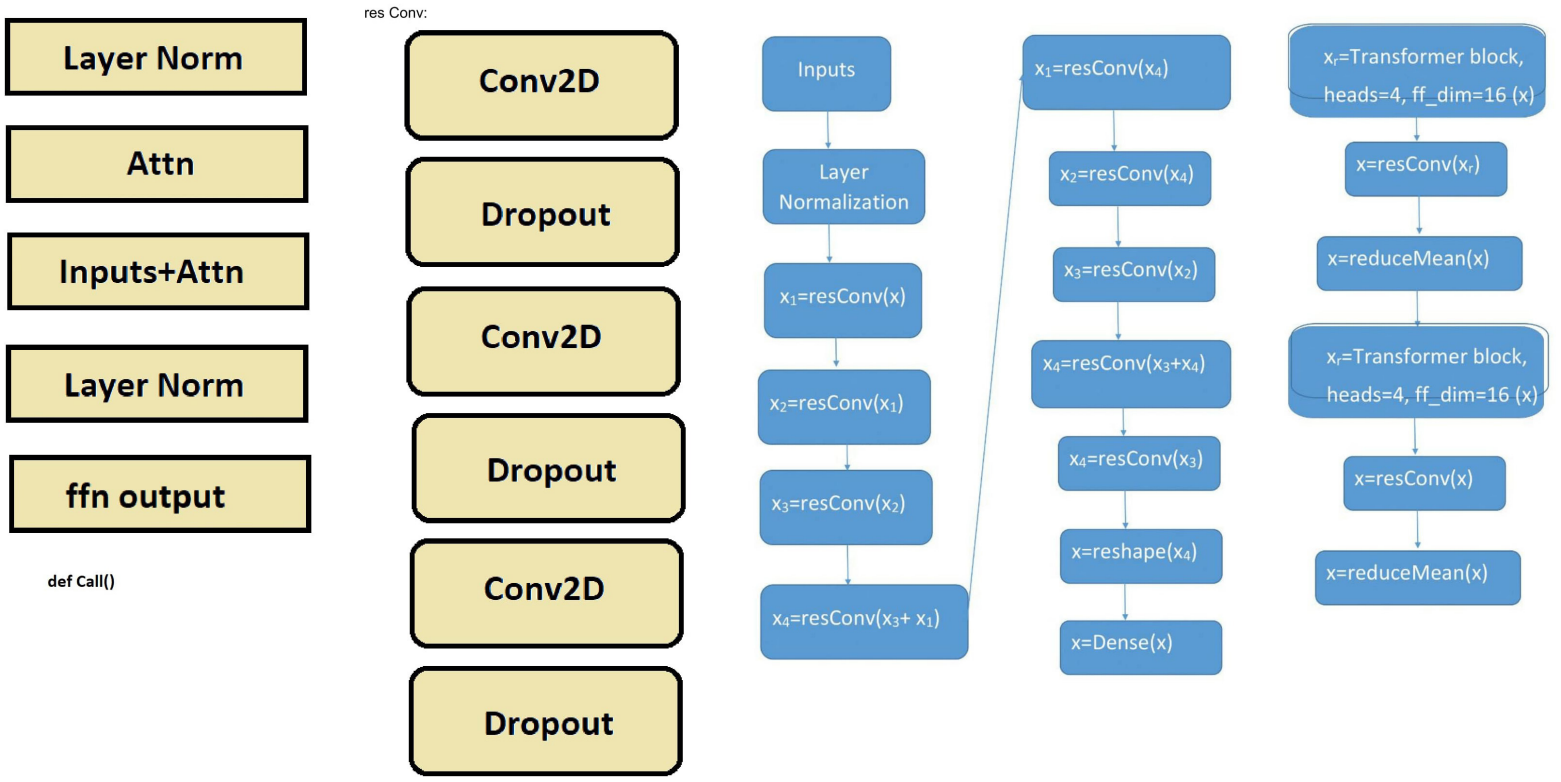
Transformer-based architecture with residual convolution blocks.

We create and encode patches. It is also possible to create multiple layers of the Transformer block. There is a Layer normalization 1. Then the authors recommend creating a multi-head attention layer, Skip connection 1, adding Layer normalization 2, MLP, and Skip connection 2. Figure 3 provides a detailed visual representation of this architecture, where each layer is clearly structured and connected. The yellow blocks illustrate the core operations (e.g., attention, normalization, feedforward) used within the blue flow diagrams representing the transformer and residual convolutional blocks.

One may create a [*batch*_*size*_, *projection*_*dim*_] tensor, add MLP, classify outputs, and create the Keras model. Then, the authors fill the train tensor-board and the validation tensor-board; if the best accuracy is less, the best loss value for all models is selected. The authors initialize the best as -infinity for custom metric and accuracy in this work. Those are three metrics: 0 for profit, 1 for accuracy, and 2 for loss.

We use sensor boards in the transformer networks model. We stop the training process if no metric is improving.

To optimize the negative log-likelihood loss importing from transformers, we employed AdamW (Loshchilov and Hutter, 2019).

### 3.5 SVM-self attention mechanisms

Support Vector Machines (SVM) are supervised learning models widely used for classification tasks, particularly in high-dimensional spaces where clear class separation is crucial. Traditional self-attention mechanisms in deep learning compute attention scores solely based on feature similarity but do not explicitly optimize for class separability. To address this limitation, we propose an SVM-enhanced attention mechanism that embeds the margin-maximization principle of SVMs directly into the attention computation. By incorporating SVM constraints, this approach ensures that feature selection is influenced not only by input relevance but also by the need to maximize the decision margin between different classes, improving EEG classification by refining class boundaries.

The proposed SVM-enhanced attention mechanism modifies the standard self-attention by enforcing margin constraints that refine attention weight computation, ensuring optimal class separation. Unlike conventional attention, which assigns weights based only on feature similarity, our approach integrates SVM optimization to refine decision boundaries and improve classification accuracy. The computed attention weights play a dual role: capturing input relevance while enforcing inter-class margin constraints to optimize class separability. The traditional self-attention mechanism computes attention scores using queries *Q*, keys *K*, and values *V* as follows:


$$\text{Attention}\left(Q,K,V\right)=\text{softmax}\left(\frac{QK^{T}}{\sqrt{d_{k}}}\right)V$$

In the SVM self-attention mechanism, the attention weights *A* are computed by solving the following optimization problem:


$$\underset{A}{\text{minimize}}\;\frac{1}{2}||A|{|}^{2}+C\overset{n}{\underset{i=1}{\sum }}\xi_{i}$$

Subject to the constraints:


$$y_{i}\left(A\cdot \phi \left(x_{i}\right)+b\right)\geq 1-\xi_{i},\;\xi_{i}\geq 0$$

Where:

- ϕ(*x*_*i*_) is the feature representation obtained from the transformer encoder,

- *y*_*i*_ represents the class labels,

- ξ_*i*_ are the slack variables,

- *C* is the regularization parameter.

**Figure 4:**
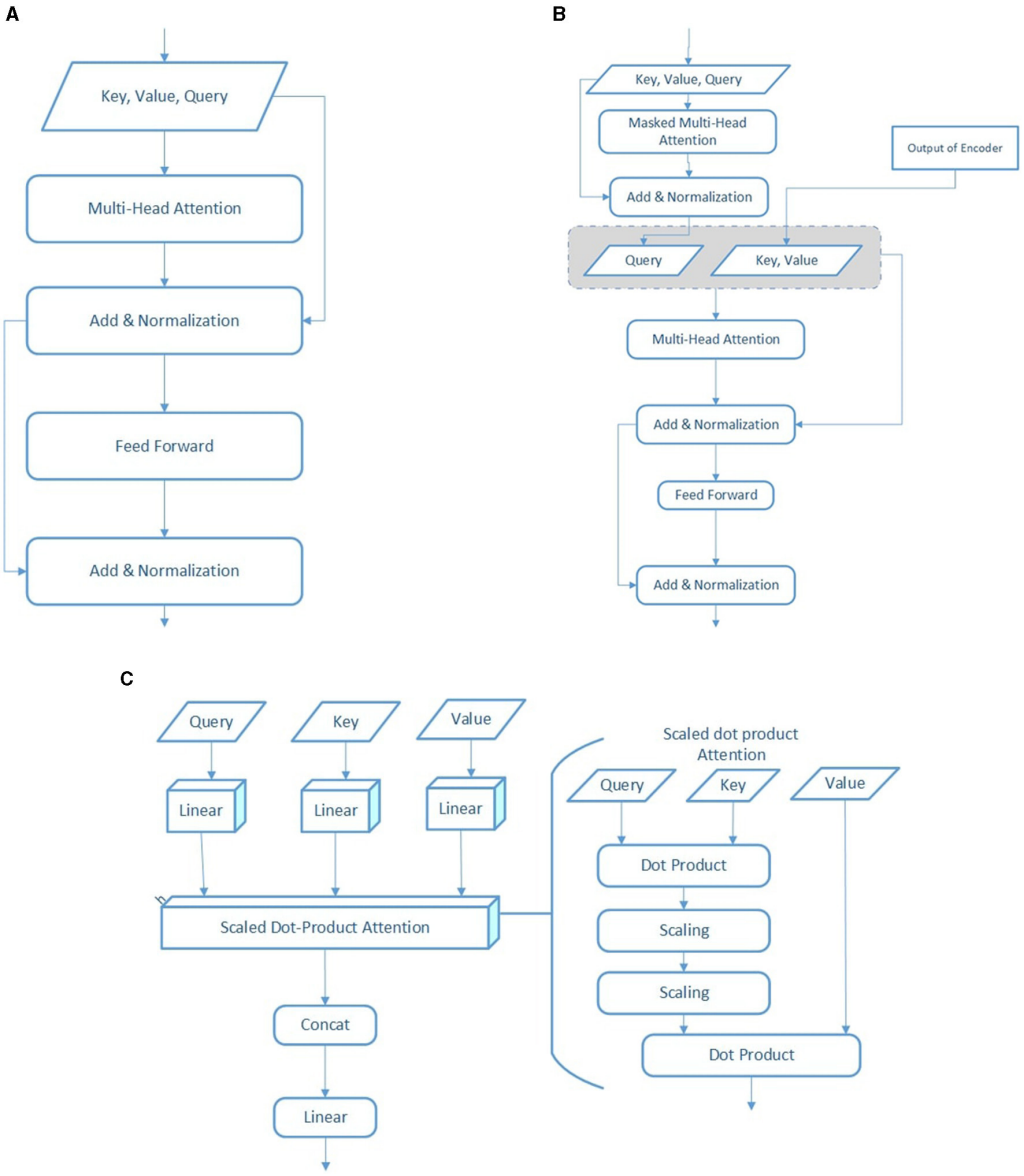
Illustration of transformer encoder/decoder module. **(A)** Transformer-encoder module. **(B)** Complete transformer architecture, highlighting the transformer decoder module. **(C)** Scaled Dot-Product attention and multi-head attention mechanisms.

Figure 4 illustrates the key components of the transformer architecture used in our model. Figure 4a shows the transformer encoder, which applies multi-head self-attention and feedforward layers to the input sequence. Figure 4b presents the decoder structure, incorporating masked self-attention for target inputs and cross-attention to encoder outputs. Figure 4c details the scaled dot-product and multi-head attention mechanisms that underlie both encoder and decoder computations. Several blocks in the figure include two outgoing arrows to represent parallel processing paths—typically one leading to a residual connection and the other to the next operation in the sequence. These paths are sequentially combined according to the standard transformer flow. In our framework, SVM-enhanced attention is incorporated within the encoder to optimize attention weights for both relevance and class separability in EEG classification tasks.

With the integration and model architecture detailed, we now reflect on the implications and potential impact of our proposed method.

### 3.6 Proposed hybrid model: SVM-enhanced attention for motor imagery EEG classification

**Figure 5:**
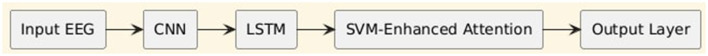
Architectural flow of SVM-enhanced attention mechanism.

The overall structure of the proposed hybrid model is illustrated in Figure 5. The model processes raw EEG signals through a sequence of modules, beginning with convolutional layers for spatial feature extraction, followed by LSTM layers to model temporal dependencies. These representations are then refined by an SVM-enhanced attention mechanism before being passed to the final output layer for classification. This layered integration is designed to combine spatial, temporal, and discriminative learning in a unified architecture.

The proposed model integrates convolutional neural networks (CNN), long short-term memory (LSTM) layers, and a novel SVM-enhanced attention mechanism to improve motor imagery EEG classification in brain-computer interface (BCI) applications. This architecture is designed to leverage the complementary strengths of CNN and LSTM while refining class separability through an SVM-driven attention mechanism.

CNN layers are employed to extract spatial features from EEG signals by capturing localized activation patterns across different electrodes. These layers are particularly effective in learning spatial dependencies within EEG data, enhancing the model's ability to distinguish motor imagery tasks. Subsequently, LSTM layers are incorporated to model temporal dependencies, ensuring that sequential relationships in the EEG time series are effectively captured. While CNN layers focus on spatial representation, LSTM layers ensure that relevant temporal information is preserved.

The SVM-enhanced attention mechanism is introduced at the final stage of feature processing to refine class boundaries by incorporating the margin-maximization principle of SVM into the self-attention layer. Unlike traditional self-attention, which assigns importance weights based on feature similarity, the proposed mechanism enforces SVM constraints to ensure that the learned feature representations are not only relevant but also optimally separated in the decision space.

The modified self-attention mechanism follows the standard attention computation:


$$\text{Attention}\left(Q,K,V\right)=\text{softmax}\left(\frac{QK^{T}}{\sqrt{d_{k}}}\right)V$$

In contrast to this softmax-based attention mechanism, our proposed SVM-enhanced attention replaces the softmax operation with weights *A* obtained through margin-based optimization. Specifically, we formulate an SVM-inspired objective that encourages the learned attention weights to maximize the separability between motor imagery classes:


$$\begin{array}{l} \underset{A}{\text{minimize}}\;\frac{1}{2}||A|{|}^{2}+C\overset{n}{\underset{i=1}{\sum }}\xi_{i}\;\text{subject to}\;y_{i}\left(A\cdot \phi \left(x_{i}\right)+b\right)\\ \;\geq 1-\xi_{i},\;\xi_{i}\geq 0 \end{array}$$

In this formulation:

- ϕ(*x*_*i*_) are the encoder-derived features (analogous to keys/queries), - *y*_*i*_ are class labels, - ξ_*i*_ are slack variables allowing soft margins, - *C* regulates the trade-off between classification error and margin size.

The optimized weights *A* replace the softmax attention scores and are used to modulate *V*, prioritizing class-separating features. This makes the attention mechanism not only context-aware but also class-discriminative.

To implement the proposed SVM-augmented attention in a differentiable manner, we reformulate the original constrained optimization as a smooth, unconstrained objective using a differentiable hinge loss. Specifically, we approximate the slack-variable-based constraint using the soft hinge function:


$$L_{\text{SVM}}=\frac{1}{2}||A|{|}^{2}+C\overset{n}{\underset{i=1}{\sum }}\max\left(0,1-y_{i}\left(A\cdot \phi \left(x_{i}\right)+b\right)\right)$$

Here, *A* represents the learned attention weights, ϕ(*x*_*i*_) are the feature representations (e.g., output of encoder or projection of query/key vectors), *y*_*i*_∈{−1, 1} are class labels, and *C* is the regularization parameter controlling the margin. This loss is fully differentiable and integrated into the computational graph, allowing gradient flow through *A* using standard backpropagation. The PyTorch autograd engine handles the gradient computation without requiring an external quadratic programming solver.

However, in our SVM self-attention, the attention weights *A* are determined by solving an optimization problem that maximizes the decision margin:


$$\underset{A}{\text{minimize}}\;\frac{1}{2}||A|{|}^{2}+C\overset{n}{\underset{i=1}{\sum }}\xi_{i}$$

subject to the constraints:


$$y_{i}\left(A\cdot \phi \left(x_{i}\right)+b\right)\geq 1-\xi_{i},\;\xi_{i}\geq 0$$

where:

- ϕ(*x*_*i*_) represents the transformed feature embeddings from the transformer encoder,

- *y*_*i*_ denotes the class labels,

- ξ_*i*_ are slack variables allowing for a soft-margin SVM formulation,

- *C* is a regularization parameter controlling the trade-off between margin maximization and misclassification penalties.

Through this formulation, the attention mechanism prioritizes features that contribute to class separability, ensuring that feature vectors belonging to different motor imagery classes are positioned with a maximized margin in the latent space.

In this hybrid framework, CNN and LSTM modules serve as feature extractors, while the SVM-enhanced attention module acts as a feature refiner to optimize class discrimination. This integration effectively mitigates issues of feature overlap and poor separability common in EEG-based classification tasks.

By embedding SVM principles directly into the self-attention layer, this model ensures that attention weight computation aligns with optimal class separation rather than mere feature relevance. The inclusion of SVM constraints enforces a geometric separation of class boundaries, thereby reducing misclassification errors and improving EEG decoding performance. This novel integration of CNN, LSTM, and SVM-enhanced attention results in a more robust and interpretable EEG classification framework suitable for real-time BCI applications.

## 4 Results

We conducted a comparative evaluation of the proposed SVM Self-Attention mechanism against several hybrid models using subject-independent evaluation, where models are tested on BCI IV 2a subjects not seen during training (LOSO protocol). The analysis focused on classification accuracy across four key motor imagery EEG datasets. Tables 1–3 present the CNN-LSTM Network test results on the BCI IV 2a, Weibo, and BCI IV 2b datasets, respectively. In these tables, the left column displays the structural hyperparameters explored during the experiments, while the first column highlights the ConvNet architecture that achieved the best performance on each dataset. This consistent format allows for a clear comparison of the network's performance across different datasets, with each table showing the results from a different dataset.

**Table 1:** Training and test accuracy (%) for BCI IV 2a using CNN+LSTM.

**Subject**	**CNN depth / Kernel size**	**Validation Acc**.	**Test Acc**.
1	(3,6,9,12,15) / (1,24)	0.71	0.75
2	(3,6,9,12,15,18,21) / (1,40)	0.57	0.56
3	(3,6,9,12,15) / (1,40)	0.55	0.53
4	(3,6,9,12,15) / (1,8)	0.90	0.92
5	(3,6,9,12,15,18) / (1,8)	0.84	0.80
6	(3,6,9,12,15,18) / (1,24)	0.78	0.72
7	(3,6,9,12,15) / (1,24)	0.71	0.76
8	(3,6,9,12,15) / (1,40)	0.81	0.78
9	(3,6,9,12,15) / (1,8)	0.75	0.82
**Average**		**0.73**	**0.74**

**Table 2:** Training and test accuracy (%) for Weibo-2014 dataset using CNN+LSTM.

**Subject**	**CNN depth / Kernel size**	**Validation Acc**.	**Test Acc**.
1	(22,44,66,88,110) / (1,8)	0.75	0.81
2	(22,44,66,88,110) / (1,40)	0.59	0.66
3	(22,44,66,88,110) / (1,24)	0.94	0.91
4	(22,44,66,88,110,132) / (1,24)	0.68	0.55
5	(22,44,66,88,110) / (1,24)	0.55	0.64
6	(22,44,66,88,110,132) / (1,24)	0.53	0.64
7	(22,44,66,88,110,132) / (1,24)	0.61	0.57
8	(22,44,66,88,110) / (1,8)	0.93	0.93
9	(22,44,66,88,110) / (1,40)	0.88	0.81
**Average**		**0.72**	**0.72**

**Table 3:** Training and test accuracy (%) for BCI-dataset 2B using CNN+LSTM.

**Subject**	**CNN depth / Kernel size**	**Validation Acc**.	**Test Acc**.
1	(60,120,180,240,300) / (1,24)	0.71	0.72
2	(60,120,180,240,300) / (1,24)	0.64	0.69
3	(60,120,180,240,300) / (1,8)	0.50	0.69
4	(60,120,180,240,300,360,420) / (1,40)	0.57	0.66
5	(60,120,180,240,300,360) / (1,24)	0.62	0.56
6	(60,120,180,240,300,360) / (1,24)	0.81	0.68
7	(60,120,180,240,300,360) / (1,24)	0.91	0.81
8	(60,120,180,240,300,360) / (1,24)	0.67	0.59
9	(60,120,180,240,300,360) / (1,24)	0.74	0.78
10	(60,120,180,240,300,360) / (1,24)	0.66	0.75
**Average**		**0.68**	**0.69**

After comparing the hyper-parameters, we also compare the ConvNetopt to EEGNet architecture based on different subjects. This process helps to choose a suitable model. The advantage is to get information about the structural hyperparameter, but the algorithmic hyperparameter is unknown. Also, both weights and epoch sizes can be estimated.

While comparing ConvNetopt to EEGNet architecture based on BCI Dataset 2B shows the following sets with percentages: training -70%; validation -15% and test-15%. The validation set helps estimate each approach's epoch length in this case. Tables 4, 5 accurately classify the following locations at different subjects using the EEGNet and ConvNetopt: training, validation, and test. To evaluate the impact of the SVM Self-Attention mechanism, we compared it against CNN-LSTM and other attention-based models on the same datasets. As summarized in Tables 6, 7, the SVM Self-Attention model consistently outperformed CNN-LSTM across BCI IV 2a, Weibo, and BCI IV 2b datasets. Compared to conventional attention mechanisms such as Multi-Head Attention and CNN-Transformer Hybrid, SVM Self-Attention demonstrated superior classification accuracy, particularly in subject-independent evaluations. The improvement is attributed to its ability to refine feature representations by optimizing class separability, a limitation in traditional attention approaches.

**Table 4:** Accuracy (%) comparison of the proposed SVM-self attention model with CNN-based and transformer-based baselines on BCI IV 2a test subjects using subject-independent evaluation (LOSO protocol).

**Model**	**S1**	**S2**	**S4**	**S5**	**S6**	**S7**	**S8**	**S9**	**Avg**.	**Params**
Deep CNN + Attention	90.11	68.22	79.12	73.45	77.34	70.12	85.11	86.33	80.91	950.21k
**SVM self attention**	**82.15**	**66.23**	**73.11**	**69.22**	**77.65**	**68.12**	**83.11**	**85.12**	**77.43**	**20.05M**
CNN-transformer hybrid	60.12	57.22	56.45	58.12	61.23	59.12	62.33	63.23	59.77	3.00M
Multi-head attention	61.13	58.33	57.22	59.44	62.14	61.12	63.44	65.12	61.03	1.02M
C[12, 24] K(3,8)	77.43	62.56	68.11	61.23	70.54	63.45	75.11	78.33	70.55	8.50M
C[24, 12] K(3, 8)	69.85	62.12	67.21	58.67	66.44	63.12	74.23	76.35	68.15	3.95M
C[48, 24, 12] K(3, 8)	72.31	55.14	70.54	62.33	75.23	61.15	78.12	79.23	70.02	2.50M
C[96, 48, 24, 12] K(3, 8)	75.22	60.11	72.11	64.22	77.41	65.01	80.54	81.14	73.14	1.40M
C[24, 12] K(3, 24)	65.41	61.23	65.12	58.45	70.13	60.23	78.15	79.23	68.35	4.10M
C[48, 24, 12] K(3, 24)	71.45	58.12	69.22	61.34	74.54	63.12	77.56	78.12	70.09	2.75M
C[96, 48, 24, 12] K(3, 24)	74.56	64.21	71.24	63.12	76.44	67.23	81.33	80.65	73.44	1.60M
C[12, 24] K(3, 8)	77.43	62.56	68.11	61.23	70.54	63.45	75.11	78.33	70.55	8.50M
C[12, 24, 48] K(3, 8)	72.11	60.32	70.11	65.21	74.66	62.54	79.24	81.11	71.96	8.60M
C[12, 24, 48, 96] K(3, 8)	74.15	62.22	72.34	66.11	78.13	65.32	82.11	84.22	74.31	8.75M
C[12, 24] K(3, 24)	68.54	63.21	66.15	62.13	73.33	61.23	76.32	80.33	70.55	8.65M
C[12, 24, 48] K(3, 24)	69.55	59.12	67.23	65.23	75.14	64.11	77.56	82.12	71.69	8.80M
C[12, 24, 48, 96] K(3, 24)	71.12	61.23	69.45	67.12	77.45	65.22	80.11	83.33	73.58	8.92M

Bold values indicate the results obtained using the proposed method presented in this study.

**Table 5:** Accuracy (%) of SVM-self attention vs. CNN and attention-based models on BCI IV 2b using subject-independent LOSO evaluation.

**Model**	**S1**	**S2**	**S4**	**S5**	**S6**	**S7**	**S8**	**S9**	**Avg**.	**Params**
**SVM self attention**	**85.12**	**68.22**	**96.55**	**79.88**	**84.33**	**81.22**	**87.44**	**88.12**	**81.47**	**20.55M**
CNN transformer hybrid	80.44	70.22	94.44	78.44	82.33	77.88	85.33	86.77	80.41	1.55M
Multi-head attention	61.13	58.33	57.22	59.44	62.14	61.12	63.44	65.12	61.03	1.02M
Deep CNN + attention	90.11	68.22	79.12	73.45	77.34	70.12	85.11	86.33	80.91	950.21k
C[24, 12] K(3, 8)	74.32	60.85	91.12	74.28	78.31	73.95	80.23	82.45	74.01	510.55k
C[48, 24, 12] K(3, 8)	75.64	61.75	92.55	76.12	78.44	74.85	82.44	83.23	75.12	275.22k
C[96, 48, 24, 12] K(3, 8)	78.42	62.23	93.54	77.19	79.66	76.88	83.45	84.34	76.77	190.12k
C[24, 12] K(3, 24)	76.11	63.88	90.66	73.99	79.77	74.14	82.12	81.98	75.53	517.90k
C[48, 24, 12] K(3, 24)	77.14	65.12	91.88	75.21	81.12	76.12	83.44	82.22	76.35	310.24k
C[96, 48, 24, 12] K(3, 24)	79.77	66.45	93.12	77.44	82.34	77.33	84.55	84.65	78.01	335.66k
C[12, 24] K(3, 8)	78.55	64.55	91.44	74.55	80.22	75.88	83.11	82.44	76.29	1.00M
C[12, 24, 48] K(3, 8)	79.88	65.22	93.55	76.22	82.11	77.15	84.33	84.12	77.61	1.03M
C[12, 24, 48, 96] K(3, 8)	80.23	66.35	94.12	77.55	83.44	78.45	85.11	85.44	78.66	1.10M

Bold values indicate the results obtained using the proposed method presented in this study.

**Table 6:** Accuracy (%) of SVM-Self Attention vs. CNN and attention-based models on Weibo dataset using subject-independent LOSO evaluation.

**Model**	**S0**	**S1**	**S3**	**S4**	**S5**	**S6**	**S7**	**S8**	**S9**	**Avg**.	**Params**
**SVM-self attention**	**68.75**	**70.00**	**84.13**	**84.75**	**85.13**	**70.00**	**71.88**	**85.38**	**56.38**	**75.88**	**21.17k**
CNN transformer hybrid	58.75	59.00	70.63	75.13	79.88	72.50	72.50	68.25	55.88	67.50	2.73M
Multi-head attention	62.50	62.50	65.00	77.88	85.13	70.25	68.63	73.75	58.13	68.88	128.8k
Deep CNN with attention	75.00	85.00	60.00	62.50	95.00	91.88	83.75	58.13	73.88	73.88	379.49k
C[16, 8] K(3, 8)	50.63	65.63	48.13	51.88	62.86	61.88	60.63	63.46	46.25	56.35	9.97M
C[32, 16, 8] K(3, 8)	54.38	67.5	72.14	71.88	74.38	68.13	65	71.88	61.65	66.13	5.06M
C[64, 32, 16, 8] K(3, 8)	72.5	69.38	68.88	74.63	81.88	75	76.25	85	53.75	72.38	2.65M
C[16, 8] K(3, 24)	58.13	62.5	47.5	55	72.5	65.63	58.63	63.63	47.5	58.19	9.97M
C[32, 16, 8] K(3, 24)	71.88	73.75	70	72.5	75.13	71.88	70	81.88	61.65	71.01	5.09M
C[64, 32, 16, 8] K(3, 24)	68.75	64.38	72.5	74.38	80.63	78.13	68.13	79.38	60.63	71.63	2.78M
C[8, 16] K(3, 8)	59.38	58.13	50.63	69.29	71.25	75.88	68.13	51.25	68.75	62.69	19.90M
C[8, 16, 32] K(3, 8)	68.75	65.63	74.29	81.88	79.38	76.25	76.25	68.13	60.63	71.88	20.17M
C[8, 16, 32] K(3, 24)	68.13	59.38	55	65.71	73.75	63.75	60.63	73.75	56.88	63.88	19.92M
C[8, 16, 32, 64] K(3, 24)	67.5	68.13	67.86	67.5	76.88	67.5	65.63	68.25	56.88	67.13	20.04M
C[8, 16, 32, 64] K(3, 8)	68.13	65	80.63	76.25	82.5	79.38	73.13	83.63	60.25	74.44	20.84M

Bold values indicate the results obtained using the proposed method presented in this study.

**Table 7:** Accuracy (%) of SVM-Self Attention vs. CNN and attention-based models on Physionet dataset using subject-independent LOSO evaluation.

**Model**	**S0**	**S10**	**S30**	**S40**	**S50**	**S60**	**S70**	**S80**	**S90**	**Avg**.	**Params**
**SVM-Self Attention**	**77.78**	**88.10**	**66.67**	**93.18**	**99.99**	**95.56**	90.48	86.47	**93.33**	**86.47**	**1.20M**
CNN Transformer Hybrid	77.78	69.05	40.48	77.27	84.44	75.56	79.48	73.81	73.81	73.81	526.76k
Multi-Head Attention	80.34	83.33	64.29	81.82	95.56	**95.56**	92.86	88.10	83.08	83.08	264.53k
Deep CNN with Attention	75.00	85.00	60.00	62.50	95.00	91.88	83.75	58.13	73.88	73.88	379.49k
C[16, 8] K(3, 8)	71.11	76.19	57.14	70.45	93.33	86.67	77.78	88.1	50	72.19	6.69M
C[32, 16, 8] K(3, 8)	73.33	76.19	45.24	72.73	91.11	91.11	86.67	95.24	57.14	72.43	3.43M
C[64, 32, 16, 8] K(3, 8)	77.78	83.33	52.38	81.82	93.33	93.33	82.22	88.1	45.24	74.42	1.77M
C[16, 8] K(3, 24)	66.67	80.95	50	77.27	91.11	93.33	86.67	90.48	57.14	73.81	6.70M
C[32, 16, 8] K(3, 24)	64.44	71.43	59.52	75	91.11	93.33	80	85.71	59.52	73.34	3.46M
C[64, 32, 16, 8] K(3, 24)	68.89	71.43	50	79.55	91.11	95.56	84.44	90.48	64.29	74.02	1.90M
C[8, 16] K(3, 8)	75.56	73.81	61.9	84.1	95.56	86.67	91.11	90.48	42.86	74.87	13.38M
C[8, 16, 32] K(3, 8)	82.22	78.57	59.52	86.36	86.67	91.11	77.78	90.48	54.76	74.75	13.66M
C[8, 16, 32, 64] K(3, 8)	73.33	76.19	50	86.36	91.11	95.56	77.78	88.1	59.52	73.57	13.71M
C[8, 16] K(3, 24)	73.33	76.19	47.62	79.55	93.33	88.89	91.11	92.86	73.81	77	13.39M
C[8, 16, 32] K(3, 24)	73.33	83.33	52.38	70.45	91.11	88.89	68.89	88.1	78.57	75.06	13.69M
C[8, 16, 32, 64] K(3, 24)	80	85.71	45.24	77.27	88.89	86.67	88.89	90.48	61.9	75.17	13.84M

Bold values indicate the results obtained using the proposed method presented in this study.

To systematically evaluate the contribution of each architectural component, an ablation study was conducted. Table 8 summarizes the results of progressively modifying the model architecture: starting from a baseline CNN-LSTM, then adding multi-head attention, replacing LSTM with a Transformer encoder, and finally introducing the SVM-enhanced attention mechanism. Each enhancement led to consistent improvements in F1-score, class separation, and sensitivity. Notably, the integration of SVM constraints into the attention mechanism resulted in the largest performance gains, highlighting its role in improving class separability and overall model robustness.

**Table 8:** Ablation study results: contribution of CNN, LSTM, transformer, and SVM-based attention.

**Model variant**	**F1-score**	**Class separation**	**Sensitivity**
CNN + LSTM	0.74	1.12	0.65
+ Multi-head attention	0.76	1.18	0.67
+ Transformer	0.78	1.22	0.71
**+ SVM-enhanced attention (proposed in this research)**	**0.83**	**1.39**	**0.78**

Bold values indicate the results obtained using the proposed method presented in this study.

Further comparison of different CNN architectures for motor imagery EEG classification is presented in Tables 6, 7. These two datasets were selected for focused transformer-based evaluation due to their contrasting properties: BCI IV 2b includes a low number of channels (3), while Physionet contains high-density EEG recordings across a large subject pool (64 channels and 109 subjects), enabling assessment of model adaptability under varying data conditions. These tables provide the performance comparison of multiple CNN-based hybrid models, including SVM-Self Attention, CNN Transformer Hybrid, Multi-Head Attention, and Deep CNN with Attention, evaluated using Leave-One-Subject-Out (LOSO) methodology.

**Figure 6:**
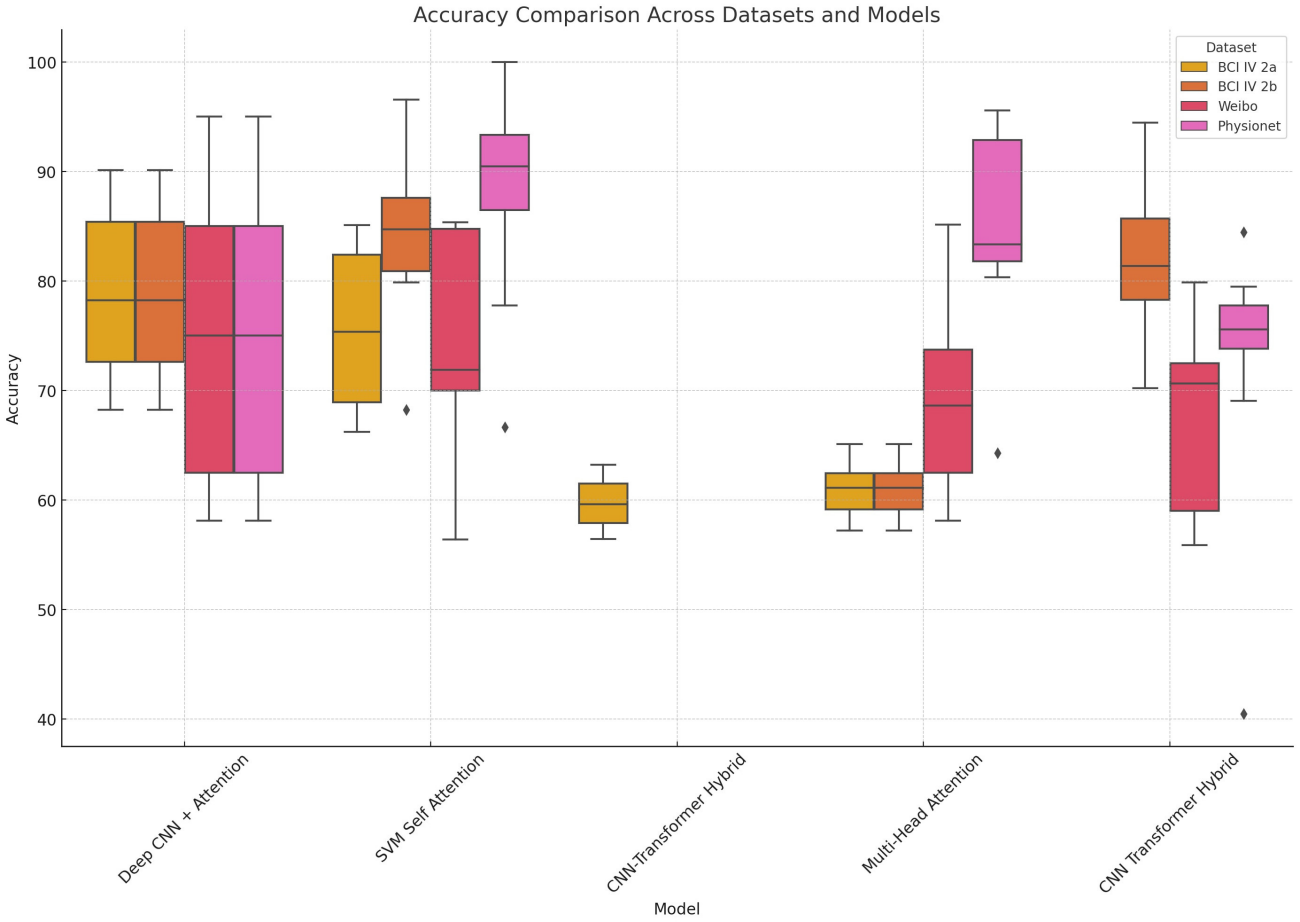
Boxplot comparison of classification accuracies for four representative models.

To assess statistical significance, we conducted one-way ANOVA tests on classification accuracy across subjects for each dataset. The results confirmed that the observed differences between models are statistically significant for BCI IV 2a (*p* = 8.56 × 10^−8^), BCI IV 2b (*p* = 7.16 × 10^−7^), and Physionet (*p* = 0.0265), while no significant difference was found for Weibo (*p* = 0.270). Figure 6 presents boxplots comparing the accuracy distributions of each model across datasets, demonstrating that our proposed SVM-Self Attention model maintains consistently high median performance and lower variance compared to other methods.

Table 6 summarizes the results for subject-independent evaluation on the Weibo dataset, while Table 7 presents results for the Physionet dataset, using the Leave-One-Subject-Out (LOSO) protocol to ensure that each subject was excluded from the training set during testing. For each subject, the best-performing models are highlighted in bold. The columns represent test results for individual subjects (S0 to S9 for Weibo, and S0 to S90 for Physionet), and the final column shows the average accuracy across all subjects. Different CNN configurations are denoted as C[2], C[12], K[3, 8], etc., representing variations in the network depth and kernel sizes. The “Params” column lists the number of parameters for each model, providing insight into the complexity of the architectures. Additionally, Table 9 compares different CNN depth configurations and their impact on output dimensionality and computation time on the Physionet dataset, demonstrating the efficiency gains achieved with reduced layer complexity.

**Table 9:** Comparison of CNN depth and EEG channel configurations on physionet dataset.

**Layer**	**EEG channels = 64**		**EEG channels = 64**	
	**CNN depth: 1–256**	**Flattened Output**	**CNN Depth: 64–448**	**Flattened output**
1	(1, 8, 64, 90)	46080	(1, 128, 1, 90)	11,520
2	(1, 16, 64, 45)	46080	(1, 192, 1, 45)	8,640
3	(1, 32, 64, 23)	47104	(1, 256, 1, 23)	5,888
4	(1, 64, 64, 12)	49152	(1, 320, 1, 12)	3,840
5	(1, 128, 64, 6)	49152	(1, 384, 1, 6)	2,304
6	(1, 256, 64, 3)	49152	(1, 448, 1, 3)	1,344
**Completion time:**		**1m 37s**	**0m 20s**	

**Figure 7:**
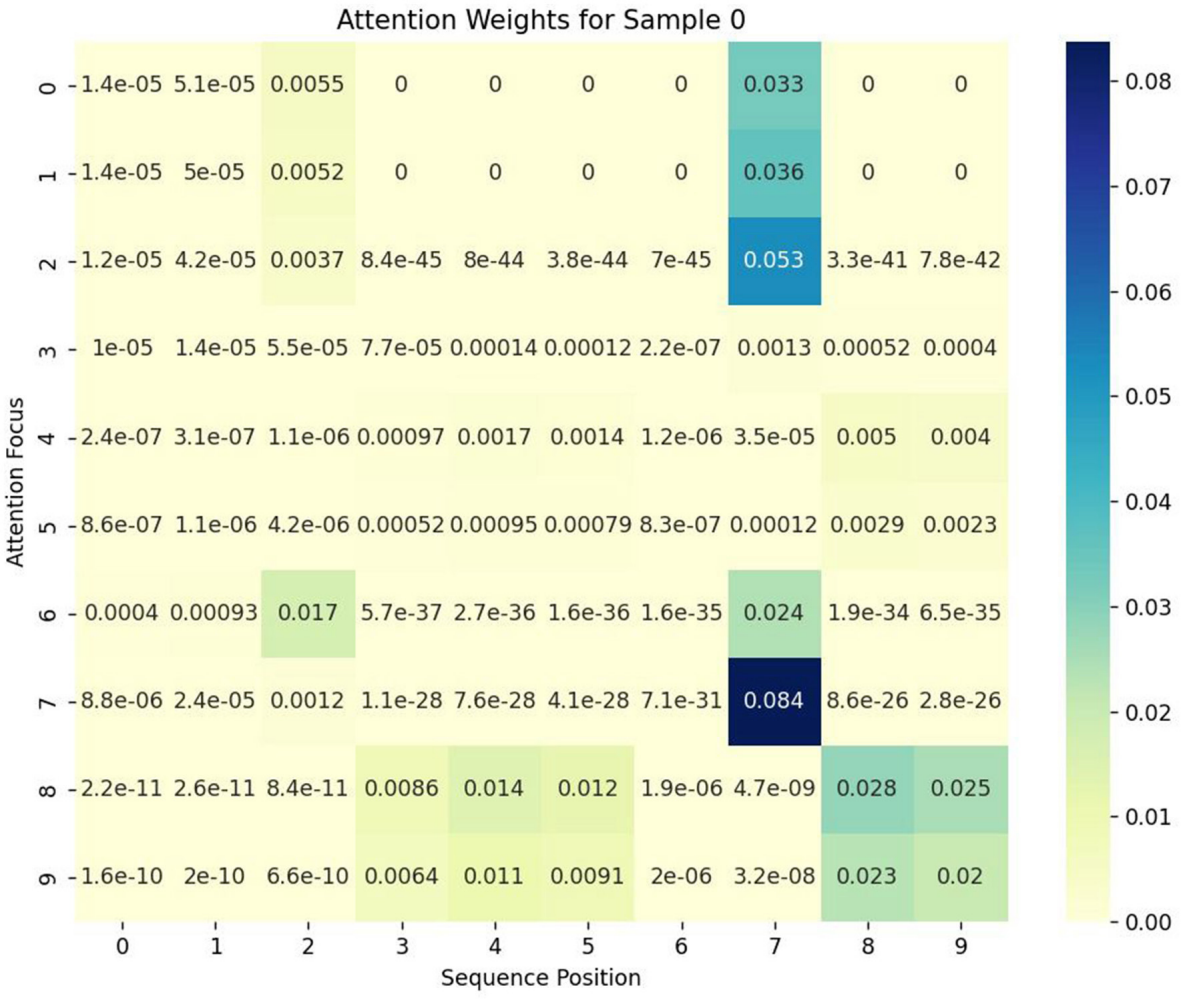
Heatmap visualization of attention weights in the SVM Self-Attention mechanism, demonstrating the model's focus on relevant EEG features across different trials.

To further illustrate the impact of SVM constraints on attention weight distribution, we present a heatmap visualization of attention weights in Figure 7. This visualization highlights how the SVM Self-Attention mechanism selectively focuses on relevant features, particularly enhancing class separability by directing attention toward more discriminative EEG signal components. The higher intensity regions in the heatmap correspond to feature positions where the SVM margin constraints exert a greater influence, reinforcing the importance of inter-class separability.

As the baseline, the results of Abibullaev et al. (2020) were used to compare, and this paper's research approach took less time than Abibullaev et al. (2020), but the results of both methods showed approximately the same accuracy.

We use three channels as the depth of CNN in the BCI IV 2b dataset. Because we made EEG channels analogically as RGB channels, we extracted features from those channels directly without creating separate CNN depth. This allowed us to end up with 1,344 flattened layer sizes instead of 49,152 for the Physionet dataset compared to work (Abibullaev et al., 2020). Considering timesteps, 448 features instead of 16,384 for the LSTM layer were used. As can be seen, more minor features resulted in faster convergence preserving similar results. These improvements in classification performance are accompanied by a reduction in computational complexity. Compared to Abibullaev et al. (2020), our CNN+LSTM model with SVM Self-Attention significantly reduces feature space dimensionality while preserving accuracy. The flattened output is reduced from 49,152 to 1,344, and training time is optimized from 1 m 37 s to 0 m 20 s.

To further contextualize the performance of our proposed SVM-Self Attention model, we compared it against recent state-of-the-art methods evaluated on the BCI Competition IV datasets. Table 10 presents classification accuracies for several benchmark architectures, including CIACNet (Liao et al., 2025), MSCFormer (Zhao et al., 2025), CLTNet (Gu et al., 2025), CTNet (Zhao et al., 2024), and EEGNet Fusion (Chowdhury et al., 2023). These models integrate advanced mechanisms such as multi-scale attention, Transformer encoders, and hybrid CNN-LSTM modules. While CIACNet achieved the highest reported accuracy of 90.05% on BCI IV 2b, our SVM-Self Attention model reached a comparable 90.48%, while also maintaining a strong result of 83.33% on BCI IV 2a-on par with or exceeding the performance of CLTNet and MSCFormer. These outcomes demonstrate that the integration of SVM-based margin optimization within the attention mechanism leads to robust generalization across different motor imagery datasets, while remaining competitive with more complex and parameter-heavy architectures.

**Table 10:** Comparison of our model with recent deep learning methods on BCI Competition IV datasets.

**Model**	**BCI IV 2a (Acc %)**	**BCI IV 2b (Acc %)**	**References**
CIACNet (CNN + attention)	85.15	90.05	Liao et al., 2025
MSCFormer (CNN + transformer)	82.95	88.00	Zhao et al., 2025
CLTNet (CNN + LSTM + transformer)	83.02	87.11	Gu et al., 2025
CTNet (CNN + transformer)	82.52	88.49	Zhao et al., 2024
EEGNet fusion (Multi-branch CNN)	74.30	84.10	Chowdhury et al., 2023
**SVM-self attention (ours)**	**83.33**	**90.48**	This work

Bold values indicate the results obtained using the proposed method presented in this study.

The CNN-LSTM network results on the Physionet dataset are provided in Supplementary materials (Supplementary Table S1). This table presents the validation accuracy (Val acc) and test accuracy (Test acc) for various CNN configurations, with each row corresponding to a different subject index (S). The CNN depth is specified as a sequence of layer sizes, and the kernel sizes are listed in parentheses [e.g., K(1,24)], indicating the dimensions used for the convolution operations. The table is organized into columns showing the subject index, CNN depth, kernel size, validation accuracy, and test accuracy, providing a structured overview of the network's performance across multiple subjects.

While this study centers on binary classification (left- vs. right-hand motor imagery), the proposed attention-enhanced CNN+LSTM model with SVM-based feature separation is architecturally compatible with multi-class classification tasks. The softmax-based output layer, cross-entropy loss function, and margin-based attention regularization can be directly scaled to handle multiple motor imagery classes, such as feet or tongue imagery, without altering the core structure. Previous studies employing attention mechanisms and CNN-LSTM hybrids for multi-class MI tasks (Zhang et al., 2020; Lawhern et al., 2018) have demonstrated that feature extraction pipelines like ours generalize well beyond binary classification when additional class labels are incorporated. Therefore, the proposed model offers a viable basis for extension to more complex BCI paradigms involving multiple control commands.

Subject-dependent analysis has low generalization capabilities. Training on the same data of the corresponding subject has high volatility considering result repeatability. However, regarding all subjects, generated data can be used for average value derivation and comparison with subject independent analysis. Below is an example of average over the best test accuracy scores which were achieve with particular CNN architectures at training time. We used the same neural network architecture as is given on Figure 2, but with new experimental activation function *abs*(*x*)*tanh(*x*) after fully connected layers. It dampens low-level fluctuations and has internal weight decaying properties.

## 5 Discussion

This study introduces a hybrid deep learning architecture that combines convolutional neural networks (CNN), long short-term memory (LSTM) layers, and an SVM-enhanced attention mechanism to improve motor imagery (MI) EEG classification in brain-computer interface (BCI) applications. The model leverages spatial feature extraction, temporal sequence modeling, and margin-based optimization to enhance classification accuracy, particularly in noisy, high-dimensional EEG data.

A key contribution of this work is the integration of SVM constraints into the attention mechanism. By embedding the margin-maximization principle, the modified attention mechanism not only captures relevant features but also improves inter-class separability. This is especially important for EEG data, which often contain overlapping patterns. As a result, the SVM-enhanced attention mechanism reduces misclassification by prioritizing features that contribute most to class differentiation, thereby improving robustness in subject-independent evaluations (Tables 6, 7).

Our model also introduces an efficient approach to CNN input design by treating EEG channels as the depth dimension, similar to RGB channels in image data. This strategy avoids the need to expand input dimensions artificially, enabling a significant reduction in the flattened layer size—e.g., from 49,152 to 1,344 in the Physionet dataset—while maintaining high classification performance. This design not only reduces computational complexity but also accelerates training, which is crucial for real-time applications.

While the integration of SVM into the attention layer introduces an additional optimization step, its complexity is constrained to a lower-dimensional attention space rather than the full feature space. Empirically, the overhead was minimal compared to the overall training time, and it was offset by faster convergence and reduced input dimensionality. Thus, the improved class separability justifies the marginal increase in computation, supporting feasibility for real-time BCI systems.

In comparative experiments across four datasets (BCI IV 2a, Weibo, BCI IV 2b, and Physionet), the CNN+LSTM architecture consistently outperformed both pure CNN and Transformer-based models. The Transformer's relatively lower performance is attributed to its need for larger datasets to capture long-range dependencies effectively, which may not be fully achievable with typical EEG data. In contrast, the LSTM component is well-suited to capturing the temporal dynamics inherent in EEG signals.

Despite its advantages, the model exhibits certain limitations. Subject-dependent analyses revealed variability in results, underscoring the need for personalization or domain adaptation strategies. Moreover, while the model was evaluated primarily on binary classification tasks, extending it to multi-class scenarios remains an important direction for future research. Addressing class imbalance and ensuring stable performance across more complex tasks are additional challenges worth investigating.

The SVM Self-Attention mechanism is particularly promising for real-time BCI applications. By enhancing class separability and suppressing noise, it supports reliable and responsive system behavior in scenarios like assistive communication, neurofeedback, and interactive control systems. Future work may explore lightweight adaptations of the attention mechanism and pruning techniques to further reduce latency and facilitate deployment in embedded environments.

Finally, Table 10 shows that our SVM-Self Attention model performs competitively or better than recent state-of-the-art methods (Liao et al., 2025; Zhao et al., 2025; Gu et al., 2025; Zhao et al., 2024; Chowdhury et al., 2023). This confirms the effectiveness of integrating margin-based optimization within deep learning frameworks for EEG decoding.

In summary, the proposed architecture effectively balances performance and efficiency by unifying CNN, LSTM, and SVM-based attention components. These findings contribute to the development of robust, interpretable, and deployable BCI systems. Future efforts should focus on improving model generalization, expanding to multi-class settings, and optimizing for real-time usage under resource-constrained conditions, while also exploring attention-based multi-modal processing advances as demonstrated in Zhao et al. (2023).

## 6 Conclusions

This study aimed to improve the classification of motor imagery (MI) EEG signals by exploring and optimizing deep learning models across four benchmark datasets: Physionet, BCI Competition IV 2a, 2b, and Weibo. A total of 109 subjects from the Physionet dataset were included, with detailed evaluation results provided in the Supplementary materials. Additional subjects from the other datasets ensured diversity and robustness in cross-dataset analysis.

The primary objective was to identify a high-performing model suitable for real-time BCI systems. Experimental results demonstrated that the CNN+LSTM hybrid architecture, especially when combined with the proposed SVM-enhanced attention mechanism, achieved competitive or superior accuracy compared to state-of-the-art methods. This model effectively captured both spatial and temporal patterns and improved class separability through margin-based attention refinement.

Transformer-based models were also evaluated, particularly on the Physionet dataset, where they produced strong results. However, the CNN+LSTM approach with SVM Self-Attention consistently outperformed them across multiple settings. These findings highlight the importance of integrating class boundary optimization directly into attention mechanisms for complex, noisy EEG signals.

Additionally, the CNN architecture introduced by Abibullaev et al. (2020) was revisited and demonstrated comparable accuracy with significantly lower computational time. This supports its use as a lightweight yet effective baseline for MI classification.

In conclusion, the proposed architecture provides a robust and computationally efficient solution for MI EEG classification, with strong potential for real-time brain-computer interface (BCI) applications.

## Data Availability

Publicly available datasets were analyzed in this study. This data can be found at: BNCI 2014-2a and BNCI 2014-2b: http://bnci-horizon-2020.eu/database, Weibo 2014: https://doi.org/10.7910/DVN/27306 and Physionet Motor Imagery EEG: https://physionet.org/content/eegmmidb/1.0.0/.

## Supplementary material

Supplementary Table S1 Classification accuracy results of multiple deep learning models across four benchmark EEG datasets (BCI IV 2a, BCI IV 2b, Weibo, and Physionet). The table contains subject-wise performance under the Leave-One-Subject-Out (LOSO) methodology and associated model parameters.

## References

[B1] Abibullaev B. Dolzhikova I. Zollanvari A. (2020). A brute-force cnn model selection for accurate classification of sensorimotor rhythms in BCIS. IEEE Access 8, 101014–101023. 10.1109/ACCESS.2020.2997681

[B2] Al-Saegh A. Dawwd S. A. Abdul-Jabbar J. M. (2021). Deep learning for motor imagery EEG-based classification: a review. Biomed. Signal Process. Control 63:102172. 10.1016/j.bspc.2020.102172

[B3] Cao L. Dong Y. Fan C. (2024). Advancing classroom fatigue recognition: a multimodal fusion approach using self-attention mechanism. Biomed. Signal Process. Control 87:105701. 10.1016/j.bspc.2023.105756

[B4] Chowdhury R. R. Muhammad Y. Adeel U. (2023). Enhancing cross-subject motor imagery classification in EEG-based brain-computer interfaces by using multi-branch CNN. Sensors 23:7908. 10.3390/s23187908 37765965 PMC10536894

[B5] Craik A. He Y. Contreras-Vidal J. L. (2019). Deep learning for electroencephalogram (EEG) classification tasks: a review. J. Neural Eng. 16:31001. 10.1088/1741-2552/ab0ab5 30808014

[B6] Gao D. Yang W. Li P. Liu S. Liu T. Wang M. (2024). A multiscale feature fusion network based on attention mechanism for motor imagery EEG decoding. Appl. Soft Comput. 151:111129. 10.1016/j.asoc.2023.111129

[B7] Goldberger A. L. Amaral L. A. Glass L. Hausdorff J. M. Ivanov P. C. Mark R. G.. (2000). Physiotoolkit, and physionet: components of a new research resource for complex physiologic signals. Circulation 101, 215–220. 10.1161/01.CIR.101.23.e215 10851218

[B8] Gu H. Chen T. Ma X. Zhang M. Sun Y. Zhao J. (2025). Cltnet: a hybrid deep learning model for motor imagery classification. Brain Sci. 15:124. 10.3390/brainsci15020124 40002457 PMC11852626

[B9] Lawhern V. J. Solon A. J. Waytowich N. R. Gordon S. M. Hung C. P. Lance B. J. (2018). EEGNet: a compact convolutional neural network for EEG-based brain-computer interfaces. J. Neural Eng. 15:56013. 10.1088/1741-2552/aace8c 29932424

[B10] Leeb R. Lee F. Keinrath C. Scherer R. Bischof H. Pfurtscheller G. (2007). Brain-computer communication: motivation, aim, and impact of exploring a virtual apartment. IEEE Trans. Neural Syst. Rehabil. Eng. 15, 473–482. 10.1109/TNSRE.2007.906956 18198704

[B11] Liang Z. Zhou R. Zhang L. Li L. Huang G. (2021). EEGFuseNet: hybrid unsupervised deep feature characterization and fusion for high-dimensional eeg with an application to emotion recognition. IEEE Trans. Affect. Comput. 29, 1913–1925. 10.1109/TNSRE.2021.3111689 34506287

[B12] Liao W. Miao Z. Liang S. Zhang L. Li C. (2025). A composite improved attention convolutional network for motor imagery EEG classification. Front. Neurosci. 19:1543508. 10.3389/fnins.2025.1543508 39981403 PMC11841462

[B13] Liu C. Huang P. (2024). DualDomain-AttenNet: synergizing time-frequency analysis and attention mechanisms for motor imagery BCI enhancement. Biomed. Signal Process. Control. 62:102697. 10.1016/j.aei.2024.102697

[B14] Liu S. Wang Z. An Y. Zhao J. Zhao Y. (2023). EEG emotion recognition based on the attention mechanism and pre-trained convolution capsule network. Knowl.-Based Syst. 265:110372. 10.1016/j.knosys.2023.110372

[B15] Loshchilov I. Hutter F. (2019). Decoupled weight decay regularization. arXiv [preprint]. arXiv:1711.05101. 10.48550/arXiv.1711.05101

[B16] Ma Y. Huang Z. Su J. Shi H. Wang D. Jia S.. (2023). A multi-channel feature fusion CNN-BI-LSTM epilepsy EEG classification and prediction model based on attention mechanism. IEEE Access. 11, 62855–62864. 10.1109/ACCESS.2023.3287927

[B17] Millan J. d. R. Rupp R. Muller-Putz G. R. Murray-Smith R. (2010). Combining brain-computer interfaces and assistive technologies state-of-the-art and challenges. Front. Neurosci. 4, 1–15. 10.3389/fnins.2010.00161 20877434 PMC2944670

[B18] Nijholt A. Tan D. Pfurtscheller G. Brunner C. Millan J. del R. Allison B.. (2008). Brain-computer interfacing for intelligent systems. Frontiers Neurosci. 23, 72–79. 10.1109/MIS.2008.41

[B19] Pfurtscheller C. Brunner J. del R Millan B. Allison B. Graimann F. Popescu B.. (1991). An EEG-based brain-computer interface for cursor control. Electroencephalogr. Clin. Neurophysiol. 78, 252–259. 10.1016/0013-4694(91)90040-B 1707798

[B20] Pichandi S. Balasubramanian G. Chakrapani V. (2024). Hybrid deep models for parallel feature extraction and enhanced emotion state classification. Sci. Rep. 14:24957. 10.1038/s41598-024-75850-y 39438562 PMC11496492

[B21] Prabhakar S. Lee S. (2022). Improved sparse representation based robust hybrid feature extraction models with transfer and deep learning for EEG classification. Expert Syst. Appl. 198:116783. 10.1016/j.eswa.2022.116783

[B22] Schirrmeister R. T. Springenberg J. T. Fiederer L. D. J. Glasstetter M. Eggensperger K. Tangermann M.. (2017). Deep learning with convolutional neural networks for EEG decoding and visualization. Hum. Brain Mapp. 38, 5391–5420. 10.1002/hbm.23730 28782865 PMC5655781

[B23] Somers B. Francart T. Bertrand A. (2018). A generic EEG artifact removal algorithm based on the multi-channel wiener filter. J. Neural Eng. 15:036007. 10.1088/1741-2552/aaac92 29393057

[B24] Sundararajan A. Pons A. Sarwat A. I. (2015). “A generic framework for EEG?based biometric authentication,” in 2015 12th International Conference on Information Technology - New Generations (ITNG) (Las Vegas, NV: IEEE Computer Society), 139–144. 10.1109/ITNG.2015.27

[B25] Tangermann M. Müller K. R. Aertsen A. Birbaumer N. Braun C. Brunner C.. (2012). Review of the BCI competition IV. Front. Neurosci. 6:55. 10.3389/fnins.2012.00055 22811657 PMC3396284

[B26] Tanwar R. Phukan O. Singh G. Pal P. (2024). Attention based hybrid deep learning model for wearable based stress recognition. Eng. Appl. Artif. Intell. 127:107391. 10.1016/j.engappai.2023.107391

[B27] Tao Y. Sun T. Muhamed A. Genc S. Jackson D. Arsanjani A.. (2021). “Gated transformer for decoding human brain EEG signals,” in 2021 43rd Annual International Conference of the IEEE Engineering in Medicine and Biology Society (EMBC) (Guadalajara: IEEE), 125–130. 10.1109/EMBC46164.2021.9630210 34891254

[B28] Wang J. Wang Z. Liu G. (2024). Recording brain activity while listening to music using wearable EEG devices combined with bidirectional long short-term memory networks. Alex. Eng. J. 88:102122. 10.1016/j.aej.2024.07.122

[B29] Wang Z. Ma Z. Liu W. An Z. Huang F. (2022). A depression diagnosis method based on the hybrid neural network and attention mechanism. Brain Sci. 12:834. 10.3390/brainsci12070834 35884641 PMC9313113

[B30] Yi W. Qiu S. Wang K. Qi H. Zhang L. Zhou P.. (2014). Evaluation of EEG oscillatory patterns and cognitive process during simple and compound limb motor imagery. PLoS ONE 9:114853. 10.1371/journal.pone.0114853 PMC4260914 25489941

[B31] Zhang D. Yao L. Chen K. Wang S. Chang X. Liu Y. (2020). Making sense of spatio-temporal preserving representations for EEG-based human intention recognition. IEEE Trans. Cybern. 50, 3033–3044. 10.1109/TCYB.2019.2905157 31021810

[B32] Zhao W. Jiang X. Zhang B. Xiao S. Weng S. (2024). Ctnet: a convolutional transformer network for EEG-based motor imagery classification. Sci. Rep. 14:20237. 10.1038/s41598-024-71118-7 39215126 PMC11364810

[B33] Zhao W. Zhang B. Zhou H. Wei D. Huang C. Lan Q. (2025). Multi-scale convolutional transformer network for motor imagery brain-computer interface. Sci. Rep. 15:12935. 10.1038/s41598-025-96611-5 40234486 PMC12000594

[B34] Zhao Y. He F. Guo Y. (2023). EEG signal processing techniques and applications. Sensors 23:9056. 10.3390/s23199056 38005444 PMC10674710

